# On the energy components governing molecular recognition in the framework of continuum approaches

**DOI:** 10.3389/fmolb.2015.00005

**Published:** 2015-03-06

**Authors:** Lin Li, Lin Wang, Emil Alexov

**Affiliations:** Computational Biophysics and Bioinformatics, Department of Physics, Clemson UniversityClemson, SC, USA

**Keywords:** binding energy, implicit solvation energy, molecular recognition, pH dependence, binding free energy

## Abstract

Molecular recognition is a process that brings together several biological macromolecules to form a complex and one of the most important characteristics of the process is the binding free energy. Various approaches exist to model the binding free energy, provided the knowledge of the 3D structures of bound and unbound molecules. Among them, continuum approaches are quite appealing due to their computational efficiency while at the same time providing predictions with reasonable accuracy. Here we review recent developments in the field emphasizing on the importance of adopting adequate description of physical processes taking place upon the binding. In particular, we focus on the efforts aiming at capturing some of the atomistic details of the binding phenomena into the continuum framework. When possible, the energy components are reviewed independently of each other. However, it is pointed out that rigorous approaches should consider all energy contributions on the same footage. The two major schemes for utilizing the individual energy components to predict binding affinity are outlined as well.

## Introduction

Many proteins carry their functions by interacting with other molecules, such as other proteins, DNA/RNA, peptides or small molecules. Having in mind that human cell is estimated to have roughly half million different proteins and on average each protein is involved in four interactions, one can appreciate the complexity of protein-protein interaction (PPI) networks. The picture becomes even more complex if one considers protein-DNA and protein-RNA interactions as well. It was demonstrated that protein-DNA recognition is a complex process utilizing base- and shape-readout mechanisms (Rohs et al., 2010) and that shape and electrostatic complementarity play equal roles for forming protein-DNA complexes (Harris et al., 2012). This fascinating area and advances made in modeling it (Schlick, 2012) will not be discussed in this review, which is focused on PPI.

PPIs are essential components of the cellular function and thus understanding the forces governing interactions within PPI networks is crucial for revealing details of cellular organization. However, modeling protein-protein recognition is not an easy task (Alexov, 2008) because of several reasons: (1) Small or large conformational changes accompany the binding process (McCammon and Robinson, 2004; Boehr et al., 2009; Csermely et al., 2010). There are no completely rigid proteins. Some proteins recognize their partner via so-termed lock-and-key mechanisms and such process typically causes small backbone changes while affecting mostly side chains. Other proteins bind via induced-fit mechanism associated with significant conformational change. (2) The protein-protein binding happens in water environment. During the binding process, the water molecules at the surfaces or in the cavities of proteins may change their positions and orientations (Yamane et al., 2008). Especially, some interfacial water molecules may be removed or added during protein-protein associations. (3) The binding may cause ionization states of some residues to alter (Alexov, 2004; Onufriev and Alexov, 2013). The titratable residues located at the interfaces will experience significant environment change, which in result may change their ionization states. Non-interfacial residues may also experience ionization state changes because of the conformational changes or because of the rearrangement of charges at the interfaces. (4) Ions are essential for stabilizing some complex structures (Freeke et al., 2010). However, many binding processes are associated with ion binding or release, i.e., the ions may not be associated with either the complex or the unbound monomers (Wang et al., 2013a). Because of the above phenomena, simulating binding process is still quite challenging.

The straightforward approach is to model explicitly all phenomena in the same protocol (Zhou and Gilson, 2009). However, this will require sampling enormously large conformational space for the biomolecules involved and the corresponding water molecules, ions, and in addition, allowing protonation changes to occur during the modeling. To reduce complexity and to allow for large-scale modeling, typical implicit methods consider water phase and biomolecules to be two distinctive homogeneous media with characteristic dielectric constants. Such an approach drastically reduces the complexity and makes the simulations much faster than with explicit model. However, this comes with the high price of losing some important atomic details. Here we outline the progress made in developing continuum methods to model molecular recognition that allow for mimicking some of the missing atomic details (Li et al., 2013a).

In the framework of continuum approaches, the binding free energy is modeled as contributions from several different energy terms. They can be broadly grouped into four classes: (a) non-bonded interactions, i.e., interactions that do not involve chemical bonds; (b) bonded interactions, typically referred as internal energies; (c) the energy term resulting from proton uptake/release upon binding and (d) the entropy change. The relative weights of these energy terms for the total binding free energy depends on the interactions being studied and the model being applied. For example, a model considering rigid binding will result in no change of the internal energy and internal entropy and this may be adequate approach for studying binding involving lock-and-key recognition (Koshland, 1995). However, a binding invoking large conformational or ionization change (Alexov, 2004), should be modeled accounting for the change of internal energy and internal entropy along with the corresponding proton uptake/release, and the contribution of these energy terms may be the major component of the binding free energy. Below we outline the physical meaning of these various energy terms and point out their treatment in the framework of continuum modeling and then discuss recent developments. It is crucial to mention that the focus of this review is the continuum framework of describing energetics of molecular recognition and what are the plausible approaches for accounting for the missing implicit details in order to predict experimentally measurable quantities.

## Electrostatic component of interaction energy between molecules

Practically each atom within a macromolecule carries a partial charge and therefore molecules participating in molecular recognition will interact with each other via electrostatic interactions. These interactions are expected to serve two major roles: to guide the molecules toward their binding mode and to provide specific interactions within the complex. Typically these electrostatic interactions are referred as Coulombic interactions, simply because of the standard approach in continuum schemes of splitting electrostatics into interactions in homogeneous media (vacuum) and the effects of water phase (Gilson and Honig, 1988). In particular, this approach is the core of so termed Molecular Mechanics Poisson-Boltzmann/Generalized Born (MMPB/GB) models (Sharp and Honig, 1990a,b; Nicholls and Honig, 1991; Im et al., 1998; Dominy and Brooks, 1999; Bashford and Case, 2000), where electrostatic component of MM energy is calculated in vacuum.

**Figure 1:**
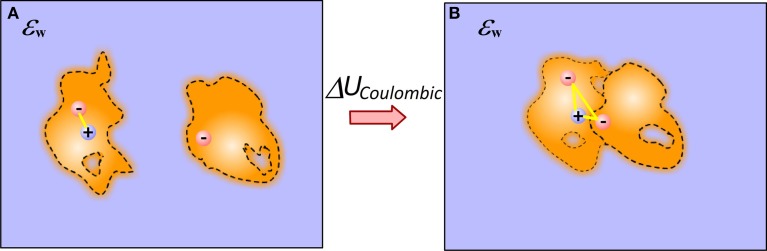
**Schematic presentation of electrostatic interactions between two molecules illustrating the origin of Coulombic component of binding energy in case of end-points approach**. **(A)** Monomers; **(B)** Complex. The inhomogeneity of molecules is shown with different intensity of the fillings and these are not the same for bound and unbound molecules. Plausible cavities and their rearrangement caused by the binding are also indicated. The internal reorganization also causes change in the internal electrostatics.

To calculate the electrostatic component of interaction energy between molecules, one considers that molecules do not interact at unbound state, while they gain some interactions in bound state (Figure 1) (Note that if the structures of bound and unbound molecules are not identical, the electrostatic component of the binding energy will also have contributions from the change of the internal electrostatics). Thus, provided the 3D structure of the macromolecular complex (and the unbound molecules) and the charges of the individual atoms, the model simply has to calculate the electrostatic interactions without accounting for the water phase. In the simplest approach, the two media approach, this is done via Coulomb law in homogeneous dielectric media (typically dielectric constant is taken either 1 (vacuum), or 2 (accounting for electronic polarizability) or some other value depending on the model). This simple approach has several consequences: it assumes that dielectric inhomogeneity present in the experiment is due to the water phase only; that there is a well-determined border between water and solutes, and that either molecules are homogeneous in vacuum or the modeling scheme includes enough representative structures to account for conformational flexibility observed in the experiment (see Figure 1).

These approximations are quite severe for the models using end-point approaches utilizing a single representative structure (or very few structures) only. The modeling can be significantly improved, in terms of mimicking the physical phenomena occurring at the binding, by considering that electrostatic interactions across molecules are not in homogeneous medium, but rather in a media which dielectric properties reflect the difference between surface bound and bulk water, between rigid and flexible regions of the molecules and presence of partially occupied water sites (at the interfaces and inside macromolecules) (Li et al., 2013b). This can be done via dielectric function, treating both the macromolecular interior and molecule-water interface in appropriate manner (Li et al., 2013b). The problem does not completely vanish even in case of having almost unlimited number of representative conformations—still considering the interfacial and bulk water to have the same properties is not physically adequate.

## Electrostatic component of solvation energy

**Figure 2:**
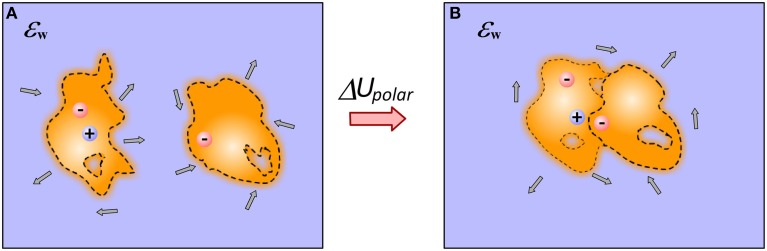
**Cartoon presentation of the details of modeling polar solvation energy in case of end-point approaches**. **(A)** Monomers; **(B)** Complex. For illustration, several water dipoles are shown in the water phase. Note that the binding changes the shape of molecules, position of charges, induces a new cavity at the interface and rearranges the internal cavities and flexibility.

Second major electrostatic component of the binding free energy is the polar solvation energy (Figure 2). In terms of continuum electrostatics it is referred as electrostatic component of solvation energy, Born solvation energy or reaction field energy (Gilson and Honig, 1988). Essentially this is the energy needed to charge an ion in appropriate solvent (such as water). Conventional approaches require that the radius of the ion is provided or in case of macromolecule, the interface macromolecule-water is given. In the last case, the electrostatic component of solvation energy is delivered either via solution of Poisson-Boltzmann (PB) or Generalized Born (GB) models.

Traditionally, these implicit methods for modeling the electrostatic properties of protein use two dielectric patterns: the entire protein is assigned low dielectric constant while the solvent (water) phase is considered as a high dielectric constant medium (Nicholls and Honig, 1991). However, this simple model describes the molecule-solvent interface as a sharp dielectric boundary between two homogeneous dielectric media. Also, it lacks the consideration of the inhomogeneous property of macromolecules and sometimes overestimates the solvation energy. Some revised models have been developed with considerations to account for conformational flexibility of macromolecules. For example, partitioning the protein into different dielectric regions based on residue side-chain flexibility (Wang et al., 2013b) and using a smooth Gaussian-based dielectric function to treat the entire system as inhomogeneous dielectric medium (Li et al., 2013c).

The early development of GB method is described in several review papers (Bashford and Case, 2000; Onufriev et al., 2000). The key component is delivering the effective Born radii. Recently empirical modifications of the original formula were proposed (Lee et al., 2002; Onufriev et al., 2002; Im et al., 2003) including development of a new approach, the GBMV2 method, where the inverse effective Born radii are evaluated via terms proportional to *r*^−4^ and *r*^−7^(Lee et al., 2003). Another modification of the standard formula was also introduced, namely a constant offset to each radius to account for the average effect of surface invagination (Mongan et al., 2007). It was demonstrated that this approach is more accurate and more efficient than GBMV2 method. Further development from the same group, involved adding information about gradients of the radii (Onufriev and Sigalov, 2011).

The above-mentioned issue of how to treat the interface between solute and water phase was investigated in a recent work emphasizing on the hydration phenomena observed in experiments. It is suggested that charge hydration asymmetry (CHA) should be introduced in the GB model. To address the problem, the CHA effect is added to the GB equation via an analytical correction. The correction quantifies the specific propensity of CHA by the charge distribution of the water model (Mukhopadhyay et al., 2012). The heterogeneity was addressed in another study to model lipid/water interface (Tanizaki and Feig, 2005). The model allows the representation of biological membranes in the form of multiple layered dielectric regions with dielectric constants that are different from the solute cavity. Predicting the solvation energy with the proposed formalism is showed a relative error of 0.17% when compared with exact finite-difference solution of Poisson equation for a transmembrane helix test system.

Another widely applied approach to calculate electrostatic component of solvation energy is to use PB formalism (Sharp and Honig, 1990a; Nicholls and Honig, 1991). Some of the commonly used PB solvers include: DelPhi (Nicholls and Honig, 1991; Rocchia et al., 2001, 2002), PB solver implemented in Amber (Wang and Luo, 2010; Wang et al., 2012), APBS (Holst et al., 2000; Baker et al., 2001; Lu et al., 2009), Charmm (Brooks et al., 2009), MIBPB (Chen et al., 2011), ZAP (Grant et al., 2001; Word and Nicholls, 2011) and many others. With exception of Gaussian-based DelPhi (Li et al., 2013b), the rest of the approaches consider two phase model: the solute is low dielectric medium, while the solvent is a high dielectric medium. The boundary between the macromolecule and the water is a sharp dielectric border and significant efforts were devoted to develop different models and definitions of molecular surface. Some of these efforts were focused on smoothing molecular surface to fill the voids not accessible to the water molecules (Gerstein and Lynden-Bell, 1993; Grant et al., 2001; Pang and Zhou, 2013), while others on determining the effective molecular surface that will result in best agreement with MD delivered solvation energy (Bates et al., 2009; Zheng et al., 2012; Onufriev and Aguilar, 2014).

Once the dielectric border between macromolecule and the water phase is generated, then the electrostatic component of the solvation energy is calculated via several approaches, one of the best in terms of accuracy being “induced surface charges” (Rocchia et al., 2002). The method of induced charges positions the induced charges on molecular surface and then calculates their interactions with the charges of the macromolecule. However, a sharp dielectric border between the solute and water phase does not account for the transition of water dielectric properties from surface bound waters to bulk waters. In addition, as illustrated in Figure 2, the binding can induce conformational changes, resulting in rearrangement of internal charges and cavities. The molecular association may result in a new cavity at the interface. All these effects are very difficult to model in the framework of canonical continuum electrostatics. However, in case of macromolecular binding, since one is interested in the change of the electrostatic component of the solvation energy only, the following scenario can be considered. The total electrostatic energy of a molecule can be calculated in unbound state (left panel in Figure 2) and in bound state with the partner charges off (right panel in Figure 2) and the difference will be the change of the electrostatic component of the solvation energy. This can be applied even in case of Gaussian-based smooth dielectric, where there is no sharp border between the solute and the water phase (Li et al., 2013b).

## Ions contribution to the binding energy

**Figure 3:**
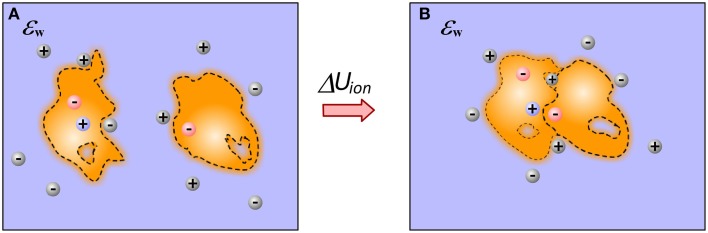
**Ions contribution to the binding**. **(A)** Monomers; **(B)** Complex. The figure illustrates a gain of ion at the interfacial cavity of bound state and reorganization of ion atmosphere caused by the binding.

Molecular recognition at physiological conditions occurs at particular non-zero ion concentration. Ions are free to move and respond to changes induced by the binding. Therefore, bound and unbound states should have different ionic “atmosphere,” resulting in different energy of interactions with mobile ions (Figure 3). Some approaches attribute this energy term directly to the electrostatic component of the solvation energy, others refer to it explicitly as “saltation” energy (Bertonati et al., 2007). In many other cases, this energy term is not taken into account, simply because it is anticipated that it has small (negligible small) contribution to the binding, although experiments have shown that it may account as much as 40% of the total binding energy (Bertonati et al., 2007).

While GB models typically account for the presence of mobile ions via Debye-Huckel screening function in the pairwise energy formula (Bashford and Case, 2000) and no much attention was paid on further developments, the treatment of ions in PB formalism attracted a lot of attention (Sharp et al., 1995; Pack et al., 1999; Rocchia et al., 2001). The traditional PB equation was expanded to include correction of finite size of ions, specific ion-ion and ion-solute interactions (Sharp et al., 1995). However, these effects are known to be important mostly in cases of macromolecules generating strong local potential (typically highly charged molecules) in presence of high ion concentration. Alternatively, one can predict non-specifically bound ions and treat them as a part of solute (Petukh et al., 2012). Adding explicit ions to the solute structure reduces the strength of the electrostatic potential and expands the applicability of standard PB approach.

Typically the contribution of ions to the electrostatic energy is calculated as the difference of electrostatic energy of the corresponding system without and with ions (Sharp et al., 1995). Other approaches explicitly calculate the excess ion concentration in the water phase and compute their interactions with the charges of the solute (Rocchia et al., 2001) (note that this methodology works only if the contribution of the ions outside the modeling volume can be neglected). In both cases, it is important to generate the Stern layer thickness and surface details according to the specificity of the protocol and type of ions being modeled. Thus, large ions should be modeled with thicker Stern layer and in general the surface of the Stern layer should be smoother as compared with the case of modeling small ions. However, applying Stern layer implies a hard surface (a sharp dielectric border) between solute and water phase, a model we were arguing against in the manuscript. In the experiments, the ions near the molecular surface compete with bound water molecules for space and binding spots. Such a competition, if not specific, is typically referred in continuum electrostatics as desolvation penalty for ions to be bound to the corresponding macromolecule. However, the magnitude of desolvation penalty depends on the dielectric property of immediate water shell surrounding the molecule. Perhaps a reasonable approach is to model the dielectric properties of the system with Gaussian-based dielectric function and to consider appropriate Stern layer according to the ion type and to calculate the difference of the electrostatic energy of the system with and without ions. Note that similarly to the considerations made for electrostatic component of solvation energy, the binding induced changes will affect the interactions with ions as well. Finally, if ion(s) is known or predicted to be bound to the macromolecules, in bound or in unbound states, it should be explicitly modeled in the computational protocol (Wang et al., 2013a).

## Non-polar component of solvation energy

**Figure 4:**
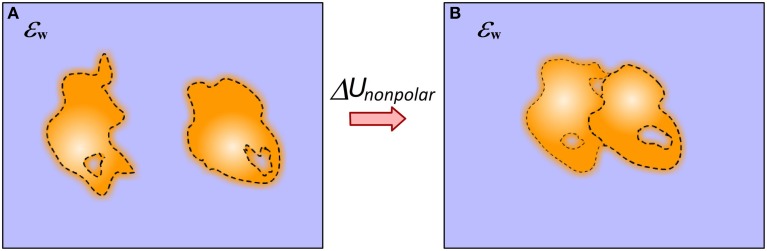
**Schematic presentation of the approach of modeling non-polar solvation energy change upon binding**. **(A)** Monomers; **(B)** Complex. The dash line indicates the smooth transition from protein interior to the water phase. This includes the layer of surface bound water molecules.

The non-polar solvation energy is the energy cost of immersing a neutral solute into the water phase and in molecular binding is the difference of immersing the complex and unbound molecules (Figure 4). In canonical approaches, when the solute and solvent are considered to be homogeneous media with sharp border between them, the non-polar solvation energy is calculated as:

(1) $$△G_{np}=\rho V+\gamma A+b$$

where *A* is the solvent accessible surface area, *V* is solvent accessible surface excluded volume, and ρ, γ, and *b* are adjustable constants (Hermann, 1972; Sitkoff et al., 1994). The ρ*V* term in the equation is based on the consideration that the non-polar solvation energy is proportional to the solvent accessible surface excluded volume (Rajamani et al., 2005; Wagoner and Baker, 2006; Chen et al., 2012). This volume based method has been proved to better fit the results from explicit solvent calculations for small proteins (Lee and Olson, 2013). However, in most of the applications, the volume term is neglected and efforts are focused on determining surface area. Many works have been done to develop fast and accurate methods of solvent accessible surface area calculation. All of these surface area calculation methods are divided into two categories: analytical methods (Connolly, 1993; Fraczkiewicz and Braun, 1998; Hayryan et al., 2005) and numerical methods (Wodak and Janin, 1980; Still et al., 1990; Eisenhaber et al., 1995; Masuya and Doi, 1995; Fraczkiewicz and Braun, 1998). Analytical methods are more accurate but also time consuming; on the contrast, the numerical methods are more efficient with the accuracy acceptable in most of the implicit solvation energy calculations.

Significant efforts were invested to optimize the parameters for the nonpolar solvation energy calculations. Different groups have suggested different *γ*-values from 5 cal/(mol*Å^2^) (Sitkoff et al., 1994) to 139 cal/(mol*Å^2^) (Ashbaugh et al., 1999) applying this approach to different problems such as: protein folding (Honig and Yang, 1994), small molecule solvation energy (Marten et al., 1996) and binding (Elcock et al., 2001). Furthermore, one can take into consideration atomic properties and develop surface tension parameters for different types of atoms. This model is called Atomic Solvation Parameters (ASP) model (Eisenberg and McLachlan, 1986; Ooi et al., 1987; Wesson and Eisenberg, 1992). Despite of these developments, still the possibility that some approaches may model the solute-water interface as a smooth transition region was not investigated. Perhaps the straightforward approach is to introduce density function (Gaussian-based density function ρ(*r*) for example), and to integrate over the volume from molecular density ρ = ρ_0_ to ρ = 0 of biomolecule. This problem bears a lot of similarity with modeling Zeta-potential in colloidal science, since it is not clear exactly where is the border between molecule attached and free water molecules.

## Protonation changes caused by binding

**Figure 5:**
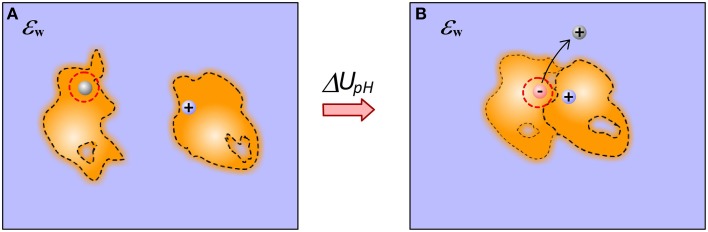
**Schematic illustration of proton release induced by the binding**. **(A)** Monomers; **(B)** Complex. The binding causes a particular acidic group, being uncharged in unbound state, to be involved in favorable interactions in the complex. This causes pKa shift, the group becomes de-protonated (ionized) and the proton is released in the water phase.

Protein-ligand bindings often accompany the changes in protonation states of the receptor and the ligand (Onufriev and Alexov, 2013) (Figure 5). A change of the ionization state of a titratable group upon the binding not only dramatically changes the electrostatic energy components, but also introduces additional correction to the binding free energy associated with addition/removal of a titratable charge at given pH. The correction is proportional to the difference between the group standard pKa and the pH of the water phase (Alexov, 2004).

Modeling the energy terms due to protonation effects induced by the binding requires predicting the ionizable states of titratable groups in bound and unbound molecules. This is quite straightforward in case of rigid body protocol or in case when 3D structures of bound and unbound molecules are available. Many methods exist for computing pKa's provided 3D structures of macromolecules (Alexov and Gunner, 1997; Georgescu et al., 2002; Gordon et al., 2005; Li et al., 2005). However, the task becomes much more complicated if the goal is to generate representative structures from bound and unbound states, since these structures should be generated allowing for different protonation states at the same time. The coupling between ionization and conformational changes is the main hurdle in many investigations focusing on binding processes as *ab-initio* docking and virtual screening (Labute, 2009; Milletti et al., 2009; Rapp et al., 2009; Petukh et al., 2013). Currently the best approach is to utilize constant-pH MD simulations (Bürgi et al., 2002; Długosz et al., 2004; Mongan et al., 2004; Machuqueiro and Baptista, 2006).

In the framework of continuum electrostatics, the ionization changes are caused by the delicate balance between unfavorable desolvation penalty and favorable interactions. These two may be due to interface formation, the most frequently occurring event, or to be caused by conformations changes propagating away from the binding interface (Alexov, 2004). In both cases, if structures of bound and unbound molecules are available, one should calculate the protonation states of titratable groups and find out which groups change their charge states from unbound to bound states. The charge appearance or deletion causes changes of all components of the electrostatic energy.

## Van der waals (vdW) energy

**Figure 6:**
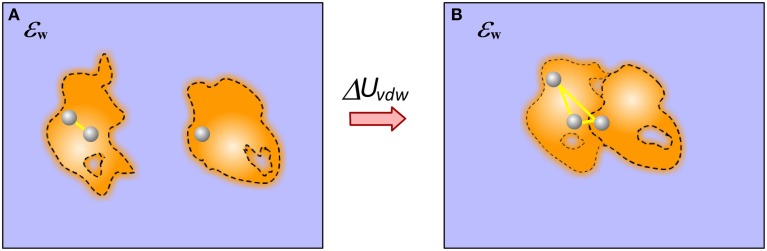
**Cartoon presentation of the vdW energy contribution to the binding**. **(A)** Monomers; **(B)** Complex. In case of end-point approach with different structures for bound and unbound states, the vdW contribution has two components: the newly formed vdW interactions across the complex interface and the change of the internal vdW interactions caused by the binding induced conformational changes.

Calculating vdW component of the binding free energy is straightforward in case of rigid binding provided the structure of the complex. In this case only vdW interactions across interface contribute to the binding. However, if flexible binding is modeled, such that the bound and unbound structures are different, the contribution of vdW energy to the binding results from both changes in internal (molecular) vdW energy and the new interactions across the interface (Figure 6).

Since vdW interactions are short-range interactions, they are typically truncated at distance larger than 10 Å. However, at short distance, the effect may be very significant mostly due to the repulsive term in case of slight atomic overlaps. Calculations involving non-relaxed structures as for example *ab-initio* docking may apply softened vdW function to tolerate small structural imperfections (Katchalski-Katzir et al., 1992). Another important point to be made is the combined treatment of vdW interactions and electrostatics. If one models electrostatics with dielectric constant different from unity, appropriate corrections should be made for vdW parameters to keep the balance and to be able to reproduce observable quantities as for example the average length of a hydrogen bond (Alexov and Gunner, 1997, 1999).

## Internal mechanical energy and entropy changes

**Figure 7:**
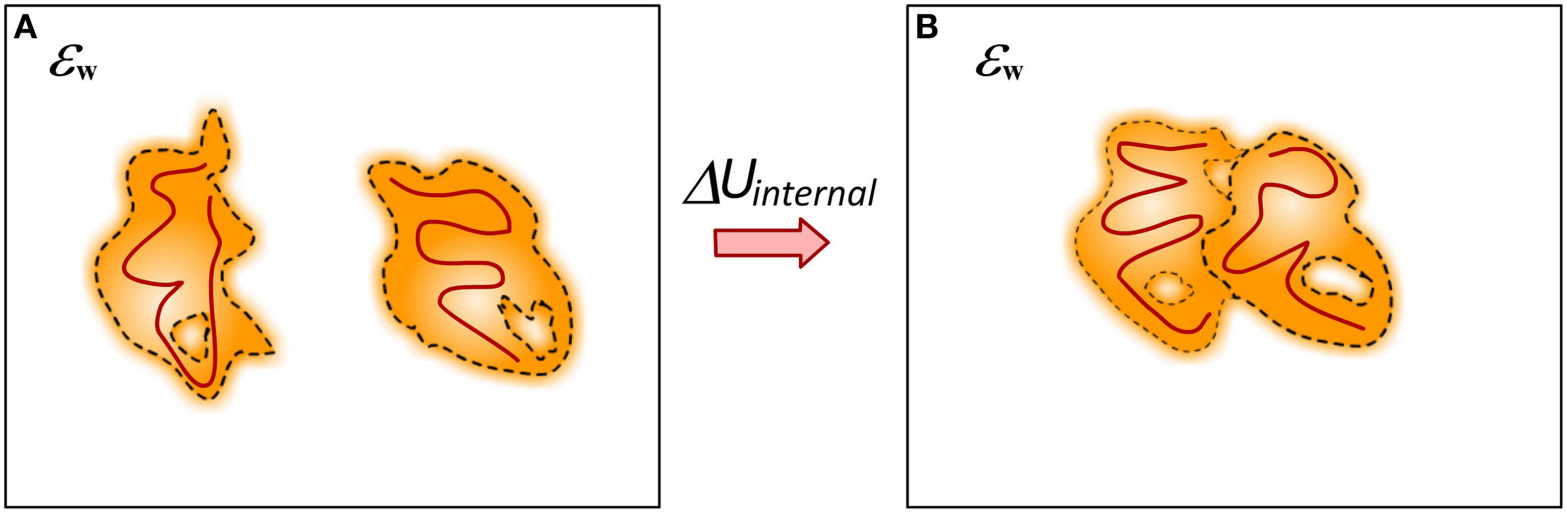
**Cartoon presentation of the conformational changes caused by the binding**. **(A)** Monomers; **(B)** Complex. Different structures of bound and unbound molecules will have different internal mechanical energy (caused by different bond angles and lengths). The internal structure changes are represented in dark red lines.

The process of binding is always associated with conformational changes in the participating molecules (Spyrakis et al., 2011) (Figure 7). In some cases the conformational changes can be small and such processes are typically referred as lock-and-key binding. In others, the binding induces large conformational changes and such processes are termed induced fit recognition (Clore, 2014; Nussinov et al., 2014). No matter how large the conformational change is, the fact that the structures of unbound and bound monomers are not identical requires the change of their internal mechanical energy to be taken into account in modeling the binding free energy. This presents a major challenge for some computational methods since small conformational changes typically result in large (over 100 kcal/mol) changes of the mechanical energy of the system. If such a change is taken directly into the energy formula, it dominates all other energy terms and results in overestimation of the binding free energy or changes of the binding free energy caused by mutations. This is the reason why many existing solutions, although considering different conformations or ensembles of conformations for bound and unbound states, still do not include mechanical energy in their energy formula (Benedix et al., 2009). Alternatively, in many approaches the unbound conformations are considered to be identical to bound, i.e., no conformational changes upon the binding, and then the change of the mechanical energy is zero (Teng et al., 2009; Kastritis et al., 2014; Li et al., 2014).

**Figure 8:**
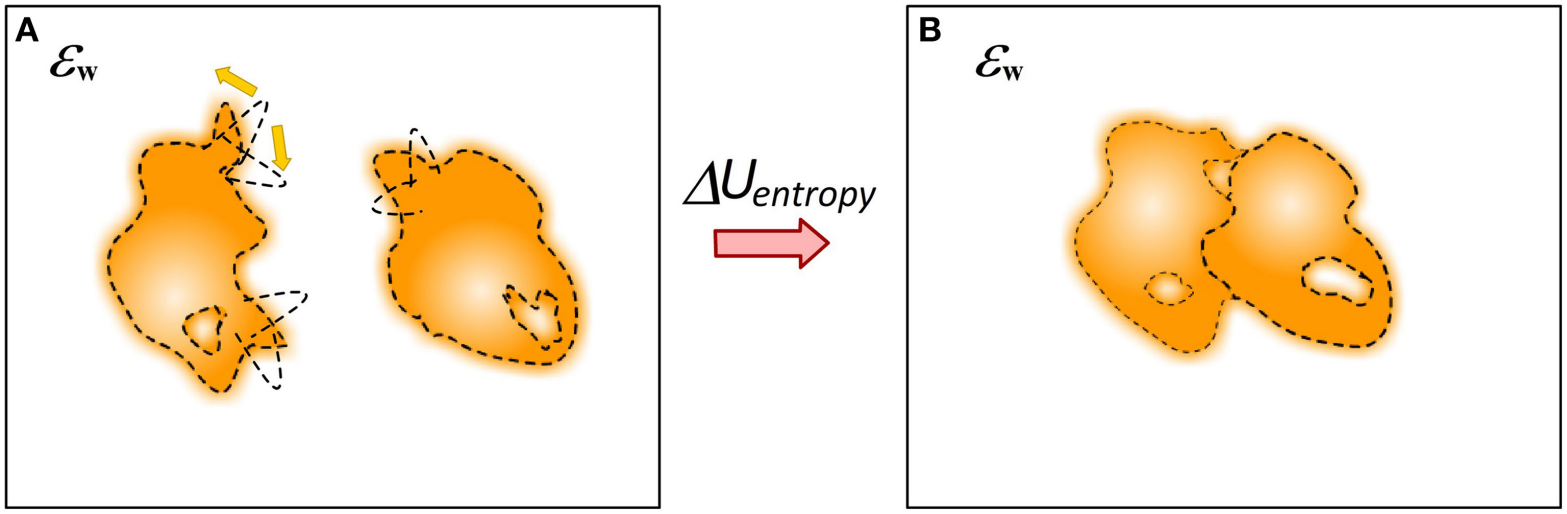
**Schematic presentation of changes of the dynamics of molecules upon the binding**. **(A)** Monomers; **(B)** Complex. The molecule can be quite flexible before binding but to adopt rigid conformation in the complex.

The question about mechanical energy and conformational changes associated with the binding cannot be considered separately from the entropy (Figure 8). The binding has profound effect of the entropy of the participating molecules, including water molecules. The simplest to account effect is the reduction of the macroscopic degrees of freedom of molecules from unbound to bound state, i.e., loss of translational and rotational entropy which in some case may account for significant fraction of the binding free energy (Silver et al., 2013). It was suggested that in many cases of protein-ligand binding the affinity is achieved by a tradeoff of essential protein-ligand contacts and at the same time allowing significant residual motion (Harpole and Sharp, 2011). Much more complicated is the evaluation of change of the internal entropy, typically referred as flexibility, of molecules upon the binding. Many approaches were developed including computing entropy change via the range of dihedrals angles changes (D'Aquino et al., 2000), probabilistic graphical models to assess Boltzmann distribution of states (Kamisetty et al., 2011) and Boltzmann-quasiharmonic method (Harpole and Sharp, 2011). The same is valid for potentially trapped water molecules at the interface of the complex (Breiten et al., 2013; Sasikala and Mukherjee, 2014). It is anticipated that if enough sampling is done, i.e., if most of relevant conformation states can be explicitly enumerated, then the balance between interaction energies and entropy changes induced by the binding may result into quite accurate predictions of the binding free energy (Wickstrom et al., 2013). The binding process may involve proton uptake/release, resulting in much more complex picture of various interconnected energy terms (Oehme et al., 2012). At the end, as pointed out by Gilson and coworkers, the energy components are interrelated and frequently the binding induces entropy-enthalpy transduction which may be the physical mechanism underlying many cases of entropy-enthalpy compensation (Fenley et al., 2012).

Macromolecular recognition involving intrinsically disordered proteins or protein fragments represents a special case of interest in terms of the interplay between enthalpy and entropy changes induced by the binding. In such cases, one or both macromolecules do not have specific 3D structure before association; however, in bound state they fold into well-defined ordered structures, resulting in a huge change of the internal entropy. It should be mentioned that this coupled binding and folding is not universal phenomena, as indicated in case of intrinsically disordered proteins with immune signaling which do not fold upon the binding (Sigalov, 2011). Another important question is the interplay between folding and binding, and which comes first. Perhaps the best approach is so termed synergistic model considering folding and binding at the same footage (Espinoza-Fonseca, 2009).

The transition from completely disordered (or unfolded) protein to completely folded one should result in large loss of entropy, which should be overcompensated by the enthalpy gain upon complex formation (Flock et al., 2014). It was argued that the change of the entropy is not as large as anticipated because even being intrinsically disordered the macromolecules retain significant fraction of their secondary structure (Chong and Ham, 2013) or repeating linear motifs (Flock et al., 2014) or some protein fragments are still disordered (Mileo et al., 2013; Hattula et al., 2014) and thus reducing the entropy cost associated with the binding. However, there must be favorable interactions occurring upon the binding that compensate for the entropy loss. These enthalpy components inducing the folding in bound state can vary from electrostatics (Chu et al., 2012), salt bridge formation (Dogan et al., 2012), polar interactions (Wong et al., 2013), phosphorylation (Nishi et al., 2013) and specific protein-membrane interactions (Lee et al., 2014). They can be studied experimentally by various techniques as mutagenesis, monitoring the binding affinity at different salt concentrations or pH and many others. Computationally, if the structure of the bound complex is available, the dominant interactions can be identified via various approaches. Overall it was found that mutations cause smaller binding energy changes in disordered protein complexes than ordered protein complexes indicating that specific interactions, although important, is less prevalent in disordered complexes (Huang and Liu, 2013). On the other hand, much more complicated is the question about the entropy change from disordered to folded state. The problem is similar to the problem of assessing the entropy contribution to protein folding (Baldwin and Rose, 2013) since it is difficult to enumerate the disordered ensemble. In case of relatively short peptides (Naqvi et al., 2014) or protein fragments (Mittal et al., 2013), one can investigate the unfolded ensemble with molecular dynamics simulations and clustering. Other approaches exploring sequence-ensemble relationships of intrinsically disordered proteins (Mao et al., 2013), rely on experimental data (Marsh and Forman-Kay, 2012; Krzeminski et al., 2013), specifically chemical shifts of backbone atoms (Terakawa and Takada, 2011; Kashtanov et al., 2012), small-angle neutron scattering (Krueger et al., 2011) and combination of experimental data and statistical analysis (Haas, 2012).

## Combining the energy terms into free energy formula

Two distinctive approaches exist of combining the abovementioned energy terms into the calculations of the binding free energy. Modeling schemes using the structure of the receptor-ligand complex, or the end-point structures (bound and unbound), or small set of representative structures for bound and unbound molecules, typically use linear interaction energy (LIE) formalism or some kind of scoring function with optimized weights (Aqvist and Marelius, 2001; Tounge et al., 2006). On the other part of the spectrum are approaches dealing with large ensemble of representative structures of bound and unbound states. If these sets are Boltzmann weighted (for example if they are generated via MD simulations), then the total free energy of the ensemble will be the arithmetic sum of the potential energy of each of the representative structures complemented with the change of the entropy caused by the binding. If the ensembles are generated by other means and are not Boltzmann weighted, then the partition function should be evaluated for each of the states (bound and unbound).

The magnitude of the abovementioned energy terms depends on many modeling parameters, including dielectric constants of solute and water, the force field charges and radii, methods of modeling molecular surface and many others. While the choice of the force field largely remains up to the investigator, the value of solute dielectric constant needs justification. Since force field parameters are optimized for explicit simulations, the approaches taking into account MM energy should calculate the electrostatic components with dielectric constant of vacuum (although some reports utilize dielectric constant of two to account for electronic polarizability of the atoms) (Kollman et al., 2000; Gouda et al., 2003). However, other approaches, which do not include MM energy or apply LIE formula may use solute dielectric constant of value larger than one or two (Kollman et al., 2000; Vicatos et al., 2009). Typically this is done to improve the correlation between predicted and experimentally available data points. However, if one is concerned about the geometry as well, a departure of the vacuum value of the solute dielectric constant should be accompanied with adjustment of vdW and MM formulas.

In the LIE formalism, the formula is constructed as a linear combination of the energy terms discussed above with adjustable weights. The optimal values of the weights are determined via benchmarking against the corresponding experimental data (Moal and Fernandez-Recio, 2012). Since each energy term contributes to the energy formula via weight coefficient, it is no longer necessary to keep the consistency of the parameters used to calculate the individual energy terms. Thus, one can obtain the internal energy term with a particular force filed and electrostatic energies with solute dielectric constant much larger than unity (Kollman et al., 2000; Vicatos et al., 2009). Typically in the LIE approaches the entropy is either not taken into account or is mimicked via some kind of approximate expression. However, since the weight coefficients are obtained by benchmarking LIE calculated energy to experimentally determined binding free energy (changes), the energies calculated with LIE are also considered to be free energies.

More rigorous approaches, as free energy perturbation (FEP) (Aaqvist, 1990), thermodynamics integration (IT) (van Gunsteren and Berendsen, 1987; Lawrenz et al., 2012), or explicit evaluation of partition function (Fisicaro et al., 1990), require all parameters and details of the modeling to be consistent. Such methods, in principle, do not require adjustable parameters optimization and benchmarking against experimental data. However, the predictions strongly depend on the ability to model all relevant conformational states, which may be computationally very demanding for binding invoking large conformational changes.

### Conflict of interest statement

The Review Editor Feng Ding declares that, despite being affiliated with the same institute as the authors Lin Li, Lin Wang and Emil Alexov and having collaborated with the authors Lin Li and Emil Alexov, the review process was handled objectively. The authors declare that the research was conducted in the absence of any commercial or financial relationships that could be construed as a potential conflict of interest.
